# Requirement of GSK-3 for PUMA induction upon loss of pro-survival PI3K signaling

**DOI:** 10.1038/s41419-018-0502-4

**Published:** 2018-04-23

**Authors:** Florian Schubert, Juliane Rapp, Prisca Brauns-Schubert, Lisa Schlicher, Kerstin Stock, Manuela Wissler, Martina Weiß, Céline Charvet, Christoph Borner, Ulrich Maurer

**Affiliations:** 1 Institute of Molecular Medicine and Cell Research, Faculty of Medicine, Albert-Ludwigs-University of Freiburg, Freiburg, Germany; 2 Spemann Graduate School of Biology and Medicine (SGBM), Albert-Ludwigs-University of Freiburg, Freiburg, Germany; 3 Faculty of Biology, University of Freiburg, Schänzlestrasse 1, 79104 Freiburg, Germany; 4 BIOSS, Centre for Biological Signaling Studies, Hebelstrasse 2, 79104 Freiburg, Germany; 5 Functional Genomics and Cancer, Institut de Génétique et de Biologie Moléculaire et Cellulaire (IGBMC), INSERM U964, CNRS UMR 7104, Université de Strasbourg, 1 rue Laurent Fries, Illkirch, 67404 France

## Abstract

Growth factor withdrawal induces rapid apoptosis via mitochondrial outer membrane permeabilization. We had previously observed that cell death of IL-3-dependent Ba/F3 cells, induced by removal of the growth factor, required the activity of the kinase GSK-3. Employing CRISPR/Cas9-mediated gene knockout, we aimed to identify pro-apoptotic GSK-3 regulated factors in this process. Knockout of either *Puma* or *Bim* demonstrated that the induction of *Puma*, but not *Bim*, was crucial for apoptosis induced by IL-3 deprivation. Thus, we aimed at identifying the GSK-3-dependent PUMA regulator. Loss of FOXO3A reduced the induction of *Puma*, while additional loss of p53 completely repressed induction upon growth factor withdrawal. A constitutively active mutant of FOXO3A, which cannot be controlled by AKT directly, still required active GSK-3 for the full transcriptional induction of *Puma* and cell death upon IL-3 withdrawal. Thus, the suppression of GSK-3 is the key function of PI3K signaling in order to prevent the induction of *Puma* by FOXO3A and p53 and thereby apoptosis upon growth factor withdrawal.

## Introduction

Growth factor signaling supports cell survival through various pathways. Thus, deprivation of growth factor ultimately results in apoptosis. The decisive step for the induction of intrinsic apoptosis is the mitochondrial outer membrane permeabilization (MOMP). This results in the release of cytochrome c and other proteins from the mitochondrial intermembrane space into the cytosol, leading to apoptosome formation, caspase activation, and apoptosis. MOMP is controlled by proteins of the BCL-2 family. While the pro-apoptotic BCL-2 proteins BAX and BAK are required for the formation of a mitochondrial outer membrane pore, their activity is induced by BH3-only proteins (PUMA, BIM, Bid, and others). MOMP is prevented by related proteins with anti-apoptotic function (like BCL-2, MCL-1, BCL-xL)^1^.

MOMP is controlled by growth factor availability, which induces various pathways promoting cell survival. A key pro-survival pathway is the PI3K/AKT signaling pathway, which can prevent MOMP and apoptosis through regulating a number of substrates. For instance, AKT was shown to phosphorylate and inactivate the transcription factor FOXO3A as well as glycogen synthase kinase-3 (GSK-3). The inactivation of both FOXO3A and GSK-3 was shown to play an important role for the pro-survival activity of PI3K/AKT signaling^2–4^. More specifically, it was shown that the suppression of FOXO3A plays an essential role for the suppression of *Puma* induction and cell death by PI3K signaling^5^.

The death promoting role of GSK-3 is instrumental for p53-mediated *Puma* induction and apoptosis: GSK-3 phosphorylates the histone acetyl transferase Tip60 (also known as KAT5), which stimulates Tip60 to acetylate p53 at K120, resulting in the transcriptional induction of *Puma* and apoptosis upon induction of p53^6^. Interestingly, GSK-3 was also shown to modulate the transcriptional activity of FOXO3A^7,8^.

In the present study, employing knockout by CRISPR/Cas9, we systematically investigated the role of GSK-3-dependent factors required for apoptosis induction by IL-3 deprivation. We show that PUMA is the main pro-apoptotic protein responsible for apoptosis in this context, and that the induction of *Puma* is mediated by a FOXO3A-, p53-, and GSK-3-dependent mechanism.

## Results

### Apoptosis induced by growth factor withdrawal requires GSK-3-dependent PUMA induction

**Fig. 1:**
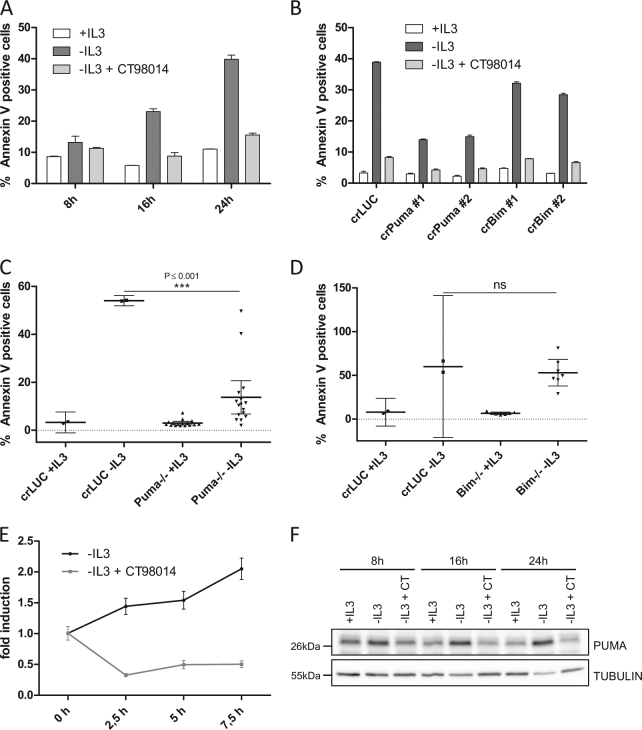
Apoptosis induced by growth factor withdrawal requires GSK-3-dependent PUMA induction. **a** Ba/F3 cells were deprived of IL-3 in the presence or absence of CT98014 (0.75 µM) and analyzed for apoptosis by Annexin V staining and flow cytometry analysis. Error bars represent SD from technical replicates. **b** Ba/F3 cells expressing CRISPR/Cas9 targeting *Luciferase* (crLUC), *Puma* (crPuma), or *Bim* (crBim) were deprived of IL-3 in presence or absence of CT98014 (0.75 µM) and analyzed for apoptosis by Annexin V staining after 18 h. Error bars represent SD from technical replicates. **c** Ba/F3 *Puma*^−/−^ single-cell clones and Ba/F3 expressing CRISPR/Cas9 constructs targeting *Luciferase* were deprived of IL-3 for 18 h and analyzed for apoptosis by Annexin V staining. Each dot represents the mean of two independent experiments analyzing an individual single-cell clone. Error bars represent 95% confidence interval from two independent experiments (*n* = 2). Significance was tested by one-way ANOVA with post hoc Tukey’s multiple comparison test. **d** Ba/F3 *Bim*^−/−^ single-cell clones and Ba/F3 expressing CRISPR/Cas9 constructs targeting *Luciferase* were deprived of IL-3 for 18 h and analyzed for apoptosis by Annexin V staining. Each dot represents the mean of two independent experiments analyzing an individual single-cell clone. Error bars represent the 95% confidence interval from two independent experiments (*n* = 2). Significance was tested by one-way ANOVA with post hoc Tukey’s multiple comparison test. ns = not significant. **e** Ba/F3 cells were deprived of IL-3 in presence or absence of CT98014 (0.75 µM) and harvested after the indicated time. The 0 h condition represents cells kept in medium with IL-3. RNA levels were analyzed by qRT-PCR with primers for *Puma* and *L32* as internal reference. Data points show relative (mRNA) *Puma* induction compared to cells kept in medium with IL-3. Error bars represent SD from technical replicates. **f** Cells from **a** were subjected to western blotting and analyzed with the antibodies indicated.

When IL-3-dependent cells such as Ba/F3 or FL5.12 cells (two murine pro B cell lines) are deprived of the growth factor, they undergo rapid apoptosis. Additional treatment with the highly selective GSK-3 inhibitor CT98014 completely blocked IL-3-withdrawal-induced apoptosis of Ba/F3 cells as observed previously^9^ (Fig. 1a). We aimed at systematically defining the pro-apoptotic factors involved in IL-3 withdrawal-induced apoptosis and at investigating their link to GSK-3. To address the role of pro-apoptotic BH3-only proteins for growth factor-withdrawal-induced apoptosis, we transduced Ba/F3 cells with the lentiCRISPRv2 system targeting either *Puma* or *Bim*. As shown in Fig. 1b, apoptosis by IL-3 deprivation was substantially reduced in Ba/F3 cells expressing CRISPR/Cas9 targeting *Puma*, while loss of *Bim* conferred only moderate protection from cell death. This effect was even more pronounced in the IL-3-dependent cell line FL5.12 (Fig. S1A). To further verify the role of PUMA in this system, clones derived from individual cells (single-cell clones) were generated from the CRISPR/Cas9-transduced cultures and cells with frameshift mutations on both *Puma* alleles or both *Bim* alleles were selected. Almost all *Puma*^−/−^ single-cell clones were strongly protected from IL-3 withdrawal-induced apoptosis (Fig. 1c) while *Bim*^−/−^ single-cell clones exhibited no statistically significant survival advantage (Fig. 1d). The protective effect of *Puma* depletion lasted at least 24 h, however, the cells committed to apoptosis at later time points. *Puma*^−/−^ Ba/F3 cells additionally treated with the GSK-3 inhibitor CT98014 showed a similar kinetic but the protective effect was more pronounced even after 40 h (Fig. S1B). Thus, the kinase activity of GSK-3 as well as the induction of *Puma* are rate-limiting for apoptosis induced by IL-3 withdrawal. The stronger anti-apoptotic effect achieved by inhibition of GSK-3 than by the loss of PUMA alone is consistent with other cell death regulatory factors being regulated by GSK-3, such as MCL-1^9^ (Fig. S1C). We next asked whether PUMA is transcriptionally regulated in a GSK-3-dependent manner upon growth factor withdrawal. Wild-type Ba/F3 cells were deprived of IL-3, treated with GSK-3 inhibitor (CT98014) and *Puma* mRNA levels were analyzed by quantitative RT-PCR. IL-3 withdrawal-induced *Puma* mRNA up to 2-fold after 7.5 h while *Puma* mRNA was reduced upon treatment with CT98014 in the absence of IL-3 (Fig. 1e). This effect was reflected by the protein levels of PUMA in Ba/F3 wt cells: PUMA was induced upon IL-3 withdrawal, but this upregulation was completely blocked by addition of CT98014 (Fig. 1f). Loss of PI3K is permitting GSK-3 activity by relieving the suppression of GSK-3 by AKT-mediated phosphorylation. Consistently, we found that the pharmacological inhibition of PI3K resulted in strong induction of PUMA (Fig. S1D).

We next generated IL-2-dependent murine primary lymphocytes, which were deprived of the growth factor. Consistent with the results obtained with IL-3-dependent cell lines, removal of IL-2 induced PUMA and apoptosis, while this was abrogated in presence of the GSK-3 inhibitor (Fig. S1E, Fig. S1F).

We therefore conclude that PUMA represents the main BH3-only protein mediating IL-3 and IL-2 withdrawal-induced apoptosis induction and that the transcriptional induction of *Puma *is dependent on GSK-3 activity.

### p53 has a minor role for GSK-3-dependent PUMA induction

**Fig. 2:**
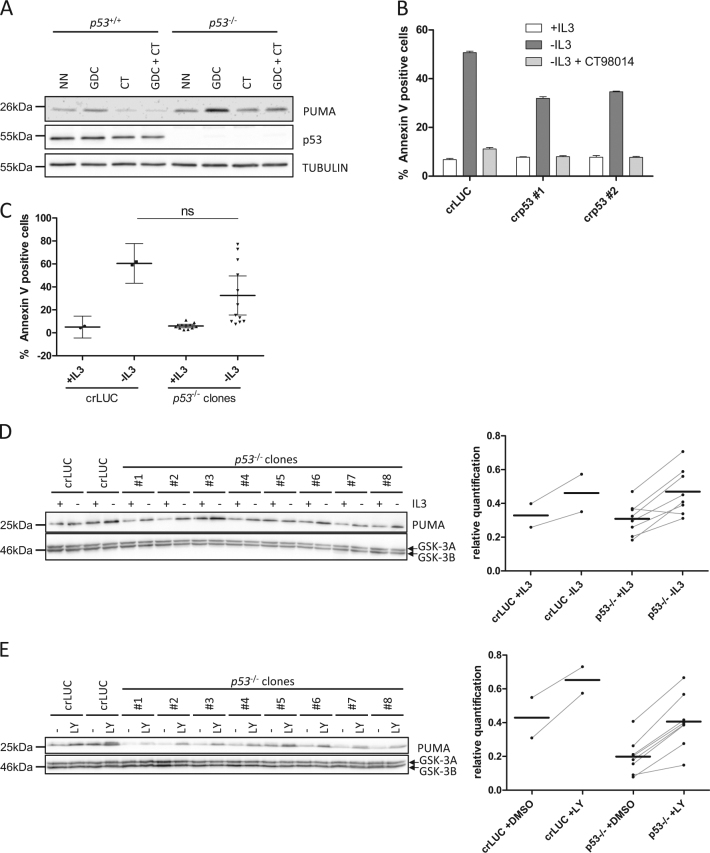
p53 has a minor role for GSK-3-dependent PUMA induction. **a** HCT116 *p53*^−/−^ or *p53*^+/+^ were treated with GDC-0941 (GDC, 10 µM), CT98014 (CT, 0.75 µM), or a combination of both for 7 h. The cells were harvested, subjected to western blotting and analyzed by the antibodies indicated. **b** Ba/F3 expressing CRISPR/Cas9 targeting *p53* or *Luciferase* were deprived of IL-3, in presence or absence of  CT98014 (0.75 µM) for 18 h and analyzed for apoptosis by Annexin V staining. Error bars represent SD from technical replicates. **c** Ba/F3 *p53*^−/−^ single-cell clones and Ba/F3 expressing CRISPR/Cas9 constructs targeting *Luciferase* were deprived of IL-3 for 18 h and analyzed for apoptosis by Annexin V staining. Each dot represents the mean of two independent experiments analyzing an individual single-cell clone. Error bars represent 95% confidence interval from two independent experiments (*n* = 2). Significance was tested by one-way ANOVA with post hoc Tukey’s multiple comparison test. ns = not significant. **d** Ba/F3 *p53*^−/−^ single-cell clones and two independent cell lines of Ba/F3 cells infected with CRISPR/Cas9 targeting *Luciferase* were deprived of IL-3 for 12 h, harvested and analyzed by western blotting by probing with antibodies as indicated. Bands were quantified using FusionCapt Advance Solo 4 16.08. PUMA levels were normalized to GSK-3 levels (loading control). **e** Ba/F3 *p53*^−/−^ single-cell clones and two independent cell lines of Ba/F3 expressing CRISPR/Cas9 constructs targeting *Luciferase* were treated with LY294002 (10 µM) or DMSO (−) for 18 h, harvested and analyzed by western blotting with the antibodies indicated. Bands were quantified using FusionCapt Advance Solo 4 16.08. PUMA levels were normalized to GSK-3 levels (loading control).

We next investigated the identity of the transcription factor, which mediates the GSK-3-dependent transcriptional induction of *Puma* upon loss of growth-factor-induced PI3K signaling. *Puma* is a crucial pro-apoptotic target of p53^10,11^. While p53 is not stabilized upon growth factor withdrawal, p53 is nevertheless able to induce some of its target genes (such as *Mdm2*) when present at very low levels. Thus, even in the absence of DNA damage, we considered p53 as a candidate for GSK-3-dependent *Puma* induction upon loss of pro-survival signaling during IL-3 withdrawal. To address the role of p53 in this context, we inhibited PI3K by GDC-0941 in HCT116 *p53*^+/+^ and *p53*^−/−^ cells in order to induce PUMA, as described before (see Fig. S1D). As shown in Fig. 2a, inhibition of PI3K-induced PUMA in *p53*^+/+^ as well as *p53*^−/−^ cells, indicating that p53 is not a major transcription factor responsible for *Puma* induction in this setting. Consistent with the data shown before, the induction of PUMA was prevented by pharmacological inhibition of GSK-3. To further investigate the role of p53, we knocked out *p53* by CRISPR/Cas9 in Ba/F3 cells, which have an intact p53 signaling pathway^6^. Upon IL-3 withdrawal, Ba/F3 cells lacking *p53* exhibited some protection from apoptosis (Fig. 2b), which was however not as strong as observed with cells lacking PUMA (see Fig. 1b). We generated *p53*^−/−^ single-cell clones and subjected them to IL-3 deprivation. We unexpectedly observed that individual Ba/F3 cell clones lacking *p53* exhibited quite some variation regarding the protection from IL-3 withdrawal-induced apoptosis. Nevertheless, on average, the protection conferred by lack of p53 was modest (Fig. 2c). Interestingly, a variation in IL-3-induced apoptosis was also observed in different single-cell clones expressing CRISPR/Cas9 targeting *Luciferase* and—of note—in wild-type Ba/F3 cells, indicating that individual clones always seems to exhibit some variation, even in the absence of previous manipulation. We therefore always analyzed a high number of clones (Fig. S2A+B). Consistent with the modest protection of *p53*^−/−^ single-cell clones from IL-3 deprivation, PUMA induction was still observed in *p53*^−/−^ single-cell clones upon IL-3 withdrawal (Fig. 2d) or PI3K inhibition by LY294002 (Fig. 2e) and was still dependent on GSK-3 (Fig. S2C). However, the quantification of PUMA expression levels revealed that, while *Puma* was still induced, the PUMA protein levels were generally somewhat reduced in *p53*^−/−^ cells (Fig. 2e, right). Together, although there is a GSK-3-dependent effect of p53 on PUMA and apoptosis induction, p53 seems not to be the main GSK-3-dependent transcription factor responsible for the induction of PUMA and apoptosis upon IL-3 withdrawal.

### FOXO3A is an important *Puma* inducer upon growth factor deprivation

**Fig. 3:**
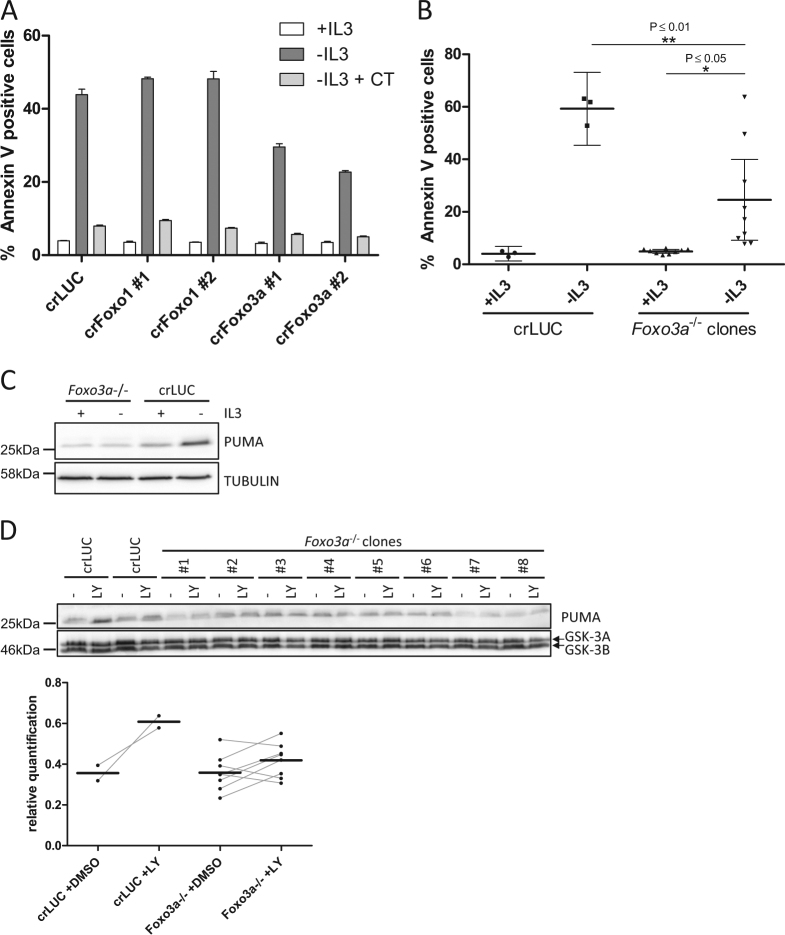
FOXO3A is an important *Puma* inducer upon growth factor deprivation. **a** Ba/F3 expressing CRISPR/Cas9 targeting *Foxo3a*, *Foxo1*, or *Luciferase* were deprived of IL-3 in the presence or absence of CT98014 (0.75 µM) for 18 h and analyzed for apoptosis by Annexin V staining. **b** Ba/F3 *Foxo3a*^−/−^ single-cell clones and Ba/F3 expressing CRISPR/Cas9 constructs targeting *Luciferase* were deprived of IL-3 for 18 h and analyzed for apoptosis by Annexin V staining. Each dot represents the mean of three independent experiments analyzing an individual single-cell clone. Error bars represent 95% confidence interval from three independent experiments (*n* = 3). Significance was tested by one-way ANOVA with post hoc Tukey’s multiple comparison test. **c** Equal cell numbers of eight different Ba/F3 *Foxo3a*^−/−^ single-cell clones were pooled. This pool and Ba/F3 expressing CRISPR/Cas9 targeting *Luciferase* were deprived of IL-3 for 9 h, harvested and analyzed by western blotting with the antibodies indicated. **d** Ba/F3 *Foxo3a*^−/−^ single-cell clones and two independent cell lines of Ba/F3 expressing CRISPR/Cas9 constructs targeting *Luciferase* were treated with LY294002 (10 µM) or DMSO (−) for 18 h, harvested and analyzed by western blotting with the antibodies indicated. Bands were quantified using FusionCapt Advance Solo 4 16.08. PUMA levels were normalized to GSK-3 levels (loading control).

To test other transcription factors for their role in inducing *Puma* upon IL-3 deprivation, we generated Ba/F3 expressing CRISPR/Cas9 targeting *Foxo1*, *Foxo3a*, and the p53 relatives *p63* and *p73*. We then subjected these cells to IL-3 withdrawal and analyzed apoptosis induction. Among the cells we tested, only *Foxo3a*-targeted cells showed a protection from IL-3 deprivation (Fig. 3a, Fig. S3A). Single-cell clones generated from these cells again showed a wide distribution of sensitivity to IL-3 withdrawal but were on average significantly protected compared to control cells (Fig. 3b). The *Foxo3a*^−/−^ single-cell clones showed a diminished capacity to induce PUMA upon IL-3 withdrawal (Fig. S3B). Because of the high variation of individual *Foxo3a*^−/−^ single-cell clones to induce PUMA, they were pooled and we observed a clear reduction of the signal for PUMA upon IL-3 withdrawal (Fig. 3d). Likewise, *Foxo3a*^−/−^ single-cell clones treated with the PI3K inhibitor LY294002 exhibited reduced *Puma* induction, as evident from the quantification of the PUMA expression levels (Fig. 3d).

### Double knockout of *Foxo3a* and *p53* fully protects cells from IL-3-induced apoptosis and prevents *Puma* induction

**Fig. 4:**
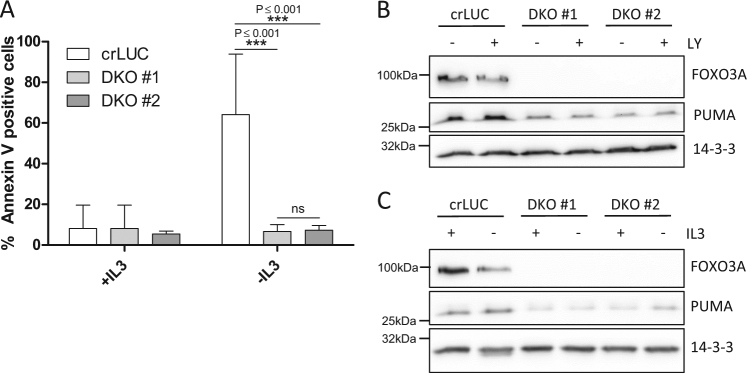
Double knockout of *Foxo3a* and *p53* fully protects cells from IL-3-induced apoptosis and prevents *Puma* induction. **a** Ba/F3 *p53*^−/−^ *Foxo3a*^−/−^ single-cell clones (DKO #1 and DKO #2) and Ba/F3 expressing CRISPR/Cas9 constructs targeting *Luciferase* (crLUC) were deprived of IL-3 for 18 h and analyzed for apoptosis by Annexin V staining. Error bars represent 95% confidence intervals from four independent experiments (*n* = 4). Significance was tested by one-way ANOVA with post hoc Tukey’s multiple comparison test. ns = not significant. ***P < 0,001. **b** The same cells were treated with LY294002 (10 µM) for 18 h or left untreated. The cells were harvested after the treatment and subjected to western blotting. The protein levels were analyzed by the antibodies indicated. **c** The same cells were deprived of IL-3 for 18 h, harvested after the treatment and subjected to western blotting.

We next addressed the combined role of p53 and FOXO3A to induce apoptosis and PUMA upon IL-3 withdrawal. We generated cells expressing CRISPR/Cas9 targeting *p53* and *Foxo3a* simultaneously and identified two single-cell clones with frameshift mutations on both alleles of each *p53* and *Foxo3a* (double knock out, DKO). As shown in Fig. 4a, both DKO clones were protected from IL-3 deprivation-induced apoptosis. In line with this, PUMA induction was absent upon treatment with LY294002 (Fig. 4b) or IL-3 withdrawal (Fig. 4c) in these cells. Thus, p53 and FOXO3A together are required for PUMA upregulation and apoptosis upon IL-3 withdrawal, possibly also compensating for each other to some extent in cells lacking either p53 or FOXO3A.

### The induction of *Puma* by FOXO3A depends on GSK-3

**Fig. 5:**
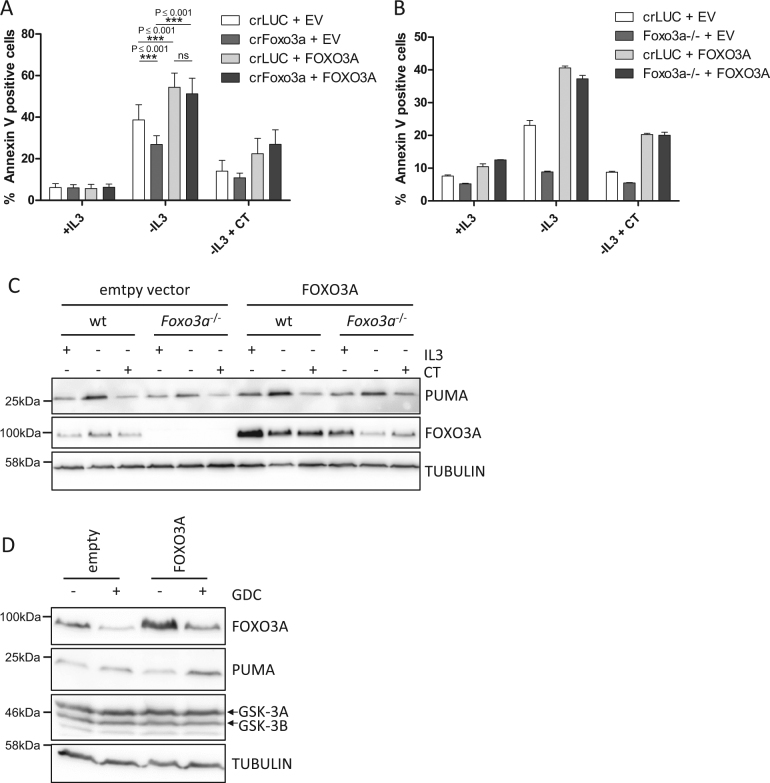
The induction of *Puma* by FOXO3A depends on GSK-3. **a** Ba/F3 cells expressing CRISPR/Cas9 constructs targeting *Luciferase* or *Foxo3a* were infected with retrovirus encoding CRISPR/Cas9-resistant human FOXO3A control retrovirus (empty vector, EV). The cells were deprived of IL-3 in the presence or absence of CT98014 (CT, 0.75 µM) for 18 h and analyzed for apoptosis by Annexin V staining. The significance was tested by one-way ANOVA with post hoc Tukey’s multiple comparison test. Error bars represent 95% confidence intervals from four independent experiments (*n* = 4). **b** Ba/F3 expressing CRISPR/Cas9 constructs targeting *Luciferase* (crLUC) or a *Foxo3a*^−/−^ single-cell clone (#4) were infected with retrovirus expressing human FOXO3A or none (empty vector, EV). The cells were deprived of IL-3 in presence or absence of CT98014 (0.75 µM, CT) for 18 h, then analyzed for apoptosis by Annexin V staining. Error bars represent SD from technical replicates. **c** Cells from **b** were harvested after 18 h and analyzed by western blotting with the antibodies indicated. **d** HCT116 *p53*^−/−^ cells were transfected by Lipofectamine2000® with vectors encoding FOXO3A or control vector (empty). Twenty-four hours after transfection, the cells were treated with DMSO or GDC-0941 (10 µM) for 5 h. The cells were harvested and analyzed by western blotting, probing with antibodies as indicated.

To confirm the major role of FOXO3A and its GSK-3 dependency in IL-3 withdrawal-induced apoptosis, we re-expressed FOXO3A with silent mutations, rendering it resistant to CRISPR/Cas9 cleavage, in a Ba/F3 bulk culture which expressed a CRISPR/Cas9 construct targeting *Foxo3a* (Fig. 5a) as well as in a *Foxo3a*^−/−^ single-cell clone (Fig. 5b). Re-expression of FOXO3A re-established apoptosis induction to a comparable level as in control cells transduced with FOXO3A in bulk culture and the single-cell clone (Fig. 5a, b). Importantly, restoring FOXO3A and thereby the competence to undergo apoptosis did not relieve the requirement for GSK-3 activity, as inhibition of GSK-3 suppressed apoptosis induced by IL-3 withdrawal in FOXO3A overexpressing cells. Thus, FOXO3A-dependent PUMA and apoptosis induction upon growth factor withdrawal requires GSK-3. Of note, the induction of apoptosis as well as the induction of PUMA in *Foxo3a*^−/−^ cells was still dependent on GSK-3 (Fig. S4A+B), confirming that the regulation of PUMA by p53 is also controlled by GSK-3^6^.

While PUMA protein levels were reduced in empty vector-transduced *Foxo3a*^−/−^ cells upon IL-3 withdrawal, PUMA induction was restored in *Foxo3a*^−/−^ cells re-expressing CRISPR/Cas9-resistant FOXO3A (Fig. 5c). Consistently, FOXO3A overexpression in HCT116 *p53*^−/−^ cells increased PUMA induction in cells treated with the AKT inhibitor GDC-0941 (Fig. 5d). We therefore conclude that FOXO3A induces *Puma* when Ba/F3 are deprived of IL-3, while p53 contributes to this induction, and that both transcription factors require GSK-3 activity for the induction of PUMA and apoptosis.

### FOXO3A requires GSK-3 activity for full transcriptional activity

**Fig. 6:**
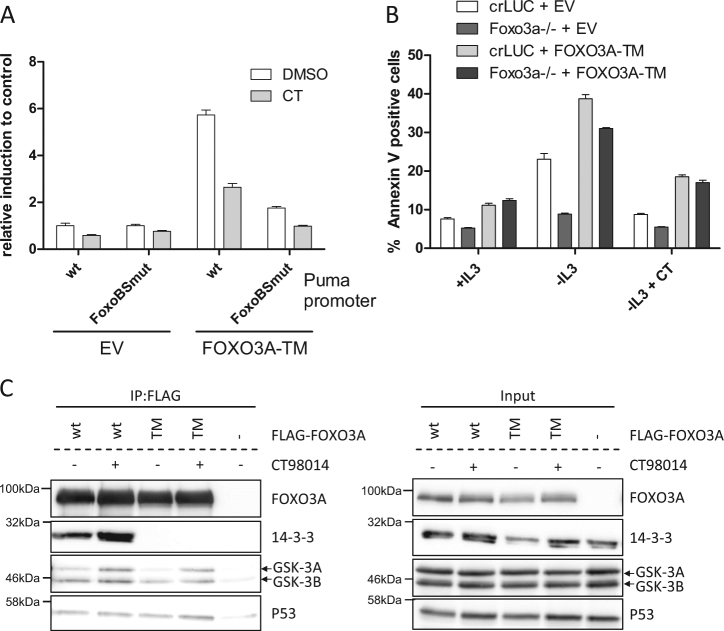
FOXO3A requires GSK-3 activity for full transcriptional activity. **a** 293T HEK cells were transfected with a reporter plasmid with Luciferase driven by a wild type (wt) or FOXO3A binding site mutant (FoxoBSmut) *Puma* promoter fragment, a Renilla reporter plasmid as internal control and a construct encoding FOXO3A-TM or empty vector. Eight hours after transfection, the cells were treated with CT98014 (CT, 0.75 µM,) or DMSO for 18 h. Luciferase activity was analyzed and normalized to Renilla activity. Error bars represent SD from technical replicates. **b** Ba/F3 cells expressing CRISPR/Cas9 constructs targeting *Luciferase* or a *Foxo3a*^−/−^ single-cell clone were infected with retrovirus expressing human FOXO3A-TM or none (empty vector, EV). The cells were deprived of IL-3+/− CT98014 (CT, 0.75 µM) for 18 h, then stained with Annexin-V-FITC and analyzed by flow cytometry. Error bars represent SD from technical replicates. This experiment was done together with the one shown in Fig. 5b, and the controls are identical. **c** HEK 293T cell were transfected with constructs encoding wild-type, FLAG-tagged FOXO3A (wt) or FLAG-tagged FOXO3A-TM (TM) or control vector (−). Eight hours later, the cells were treated with CT98014 (0.75 µM) or left untreated for 18 h. The cells were lysed and 2% of the lysate were kept as input. FLAG immunoprecipitation was performed with the remaining lysate. After washing and elution with 3xFLAG peptide, eluate and input fractions were analyzed by western blotting with the antibodies indicated.

As shown above, the pharmacological inhibition of GSK-3 was sufficient to prevent growth factor withdrawal-induced PUMA induction and apoptosis. We previously demonstrated that when p53 is stabilized by DNA damage, GSK-3 activity regulates the capacity of p53 to induce *Puma* by phosphorylating Tip60 at S86, which stimulates Tip60 to acetylate lysine 120 (K120) of p53^6^. K120-acetylated p53 then induces *Puma* which leads to apoptosis^12–14^. However, in *p53*^−/−^ cells we observed FOXO3A-dependent PUMA induction and apoptosis, which was also dependent on GSK-3 activity (Figs. 2a–c and 5d). To investigate the role of GSK-3 for the transcriptional regulation of *Puma* by FOXO3A, we generated a promoter reporter system where Luciferase expression is under control of the *Puma* promoter, which includes a binding site for FOXO3A^5^. It is well established that FOXO3A is controlled by the PI3K/AKT pathway as phosphorylation of FOXO3A residues T32, S253, and S315 by AKT results in retention of the transcription factor in the cytosol^2^. We expressed a FOXO3A-triple mutant (TM; T32A-S253A-S315A), which cannot be phosphorylated by AKT and is considered to be constitutive active, along with the *Puma* promoter reporter constructs. As shown in Fig. 6a, overexpression of FOXO3A-TM induced Luciferase expression controlled by the *Puma* promoter. This was dependent on a FOXO3A binding site in the promoter region, as mutation of the binding site for FOXO3A prevented Luciferase induction. However, when we added the GSK-3 inhibitor CT98014, the ability of FOXO3A-TM to induce Luciferase through the *Puma* promoter was substantially decreased. We therefore conclude that FOXO3A, in order to fully exhibit its transcriptional activity at the *Puma* promoter, requires not only the dephosphorylation of AKT phosphorylation sites in FOXO3A, but also the activity of GSK-3.

We next established Ba/F3 cell lines overexpressing FOXO3A-TM. We expressed FOXO3A-TM with silent mutations, mediating resistance to CRISPR/Cas9 cleavage, in a Ba/F3 bulk culture expressing a CRISPR/Cas9 construct targeting Foxo3a, or in control cells with CRISPR/Cas9 targeting Luciferase. This led to a slightly increased background of apoptosis even when IL-3 was available. However, although FOXO3A-TM is considered to be constitutively active, IL-3 deprivation was required to induce apoptosis (Fig. 6b, left). Importantly, apoptosis could be inhibited by addition of CT98014 (Fig. 6b, right), showing that FOXO3A-TM (which is not controlled by AKT) requires GSK-3 activity to induce apoptosis upon IL-3 withdrawal. As the inhibition of GSK-3 did not affect the binding to 14-3-3 proteins (Fig. 6c) or the nuclear localization of FOXO3A (Fig. S5A), the regulation of FOXO3A-mediated transcription by GSK-3 is independent of FOXO3A translocation. Interestingly, we observed an interaction of GSK-3 with FLAG-tagged FOXO3A overexpressed in 293T HEK cells (Fig. 6c). Together, the results show that activity of GSK-3 is decisive for the FOXO3A and p53-mediated PUMA induction in the absence of PI3K signaling.

## Discussion

We had previously shown that GSK-3 phosphorylates the anti-apoptotic protein MCL-1 upon growth factor withdrawal or PI3K inhibition, which regulates lymphocyte survival^9,15^. In this study, we aimed at systematically identifying the GSK-3-dependent pro-apoptotic factors, promoting apoptosis upon depletion from growth factor. We showed that PUMA rather than BIM mediates IL-3 withdrawal-induced apoptosis, although both proteins were upregulated upon IL-3 deprivation (data not shown). In line with our data, it was previously shown that PUMA and BIM are induced by IL-2 withdrawal^5,16^. Our findings also confirm a previous report that IL-3-dependent myeloid cell lines from mice lacking PUMA, but not BIM, were protected from IL-3 withdrawal-induced apoptosis^17^.

FOXO3A and p53 were both described to be important for growth factor withdrawal-induced apoptosis. FOXO3A deficiency can protect cells from cytokine withdrawal-induced apoptosis^16,18^, and FOXO3A was shown to be crucial for PUMA induction upon growth factor withdrawal^5^. It was also found that p53, FOXO and E-box-binding transcription factors share many targeted genes in response to PI3K inhibition in Rat-1 cells and a dominant-negative p53 resulted in partial resistance to apoptosis upon PI3K inhibition^19^. Likewise, HoxB8-transformed factor-dependent myeloid (FDM) cells from p53^−/−^ mice were less sensitive for IL-3 deprivation-induced cell death and showed reduced PUMA induction. In this study, FDM cells from FOXO3A^−/−^ mice showed an even higher susceptibility to cell death after IL-3 loss^20^. The same authors reported that PUMA induction was independent of PI3K, a finding which is not confirmed by our data^17,21^. Instead, we found that inhibition of the PI3K/AKT pathway upregulated PUMA in *p53*^−/−^ HCT116 cells and Ba/F3 cells.

In addition, p53 negative Ba/F3 cells were fully protected from apoptosis only when GSK-3 was inhibited, which further supports the important apoptosis-regulatory role of the PI3K/AKT pathway also in absence of p53.

We have shown that the induction of *Puma* by FOXO3A and/or p53 is dependent on GSK-3 activity. Importantly, we observed that even the transcriptional activity of a FOXO3A-TM mutant, which is considered constitutively active due to the loss of the AKT phosphorylation sites, required GSK-3. Our results suggest that the inhibition of GSK-3 is the key pro-survival function of PI3K signaling, being more important than the inactivation of FOXO3A. In line with this finding, full transcriptional activity of Foxo1/3/4 was shown to require GSK-3, as the induction of IGF-IR gene by serum starvation or AKT inhibition required both active GSK-3 and Foxo1/3/4^8^. Supporting a PI3K-dependent regulation of FOXO3A independent of the AKT phosphorylation sites, the DNA-binding and transcriptional activity of a triple S/A mutant of the Foxo homolog in *C. elegans*, DAF-16, which is considered constitutive active, was shown to be further increased by PI3K inhibition by LY294002 and Wortmannin^22^. Interestingly, promotion of FOXO3A activity to induce PUMA by GSK-3 was independent of the interaction of FOXO3A with 14-3-3 or its subcellular localization.

It is not clear however, how GSK-3 and FOXO3A cooperate. In a study of Terragni et al., upregulated genes upon inhibition of PI3K were co-regulated by FOXO3A, MITF, and USF1, with the latter two being regulated by GSK-3^23^. Another explanation for the functional interaction of FOXO3A and GSK-3 could be provided by the regulation of TIP60. We could previously demonstrate the requirement of GSK-3 for *Puma* induction by p53 in the context of DNA damage and subsequent p53 stabilization by the phosphorylation of the histone acetyl transferase Tip60 (KAT5). Increased KAT activity of Tip60 is induced by GSK-3 mediated phosphorylation, resulting in the acetylation of p53 in the DNA-binding domain at lysine 120, which enables p53 to promote the transcription of *Puma*. It is possible that GSK-3 activated Tip60 acts on chromatin or FOXO3A directly, thereby promoting transcriptional *Puma* induction^6^.

A direct phosphorylation of FOXO3A by GSK-3 would also be a possibility as to how GSK-3 directly promotes the transcriptional activity of FOXO3A, which would be consistent with our data demonstrating a cooperation of FOXO3A-TM and GSK-3 for the regulation of the *Puma* promoter.

Together, in this study, we provide evidence that *Puma* induction by FOXO3A is an important step in IL-3 withdrawal-induced apoptosis, and that the *Puma* inducing function of FOXO3A is dependent on GSK-3 activity. Thus, GSK-3 is crucial for the full activation of FOXO3A transcriptional activity when the PI3K pathway is not active. As pharmacological modulation of kinase signaling pathways is a promising strategy for cancer therapy, a more detailed understanding of the underlying mechanisms will improve defining the crucial targets.

## Materials and methods

### Reagents and antibodies

LY294002 was from Sigma-Aldrich (St. Louis, MO, USA), GDC-0941 and CT98014 were from Axon Medchem (Groningen, Netherlands), Annexin-V-FITC was generated in our lab. Annexin-V-APC (RUO) was from Becton Dickinson (Franklin Lakes, NJ, USA). 4-OHT was from Sigma-Aldrich (St. Louis, MO, USA).

IL-2 and IL-3 were from Peprotech (Rocky Hill, NJ, USA).

The following antibodies were used for western blotting: Puma (#3043) was from Prosci (Poway, CA, USA), human Puma (#12450), Foxo3a (#2497) were from Cell Signaling Technologies (Danvers, MA, USA). GSK3a/b (sc-56913), 14-3-3 (sc-1657) and NFATc1 (sc-7294) were from Santa Cruz (Dallas, TX, USA). Tubulin (MCA77G) was from Bio-Rad (Hercules, CA, USA). MCL1 (600-401-394S) was from Rockland (Limerick, PA, USA).

FLAG-M2 agarose affinity beads were from Sigma-Aldrich (St. Louis, MO, USA).

### Generation of ko cell lines using lentiviral CRISPR/Cas9

The lentiCRISPRV2 system was used to generate ko cells as described by others^24^. At least two different guide RNAs for each target were designed using crispr.mit.edu and a guide RNA targeting *Luciferase* was designed as control (see Table 1: gRNAs). The guide RNAs were cloned into the lentiCRISPRV2 plasmid (a gift from Feng Zhang, Addgene plasmid # 52961) and lentiviral particles were produced by transfection of 5 µg lentiCRISPRv2, 1.5 µg pMISSIONVSV-G (Sigma-Aldrich) and 3 µg pMISSION GAG POL (Sigma-Aldrich) with Attractene (Quiagen, Hilden, Germany) into 293T HEK cells seeded at 25% confluency in a 78-cm² culture plate the day before transfection. The morning after transfection, fresh medium was added. In the evening, 4 ml medium was added. The next day, viral supernatants were harvested, filtered (0,45 µM) and supplemented with 5 µg/ml polybrene (Sigma-Aldrich). The target cells were infected by spinfection at 400×*g* for 10 min. The following day, selection was started using 4 µg/ml puromycin (Sigma-Aldrich) for at least 3 days and until no viable cells were detected in an uninfected control. Mix cultures were tested for Cas9 cleavage by performing a Surveyor assay. The region of interest was amplified by PCR using the primers listed (Table 2: Primers). The Surveyor assay was performed according to the manufacturer (Integrated DNA Technologies). The mix cultures were used for experiments and to generate single-cell clones by limited dilution. Single-cell clones were analyzed by sequencing and clones with a bi-allelic shift in the open reading frame generating a premature STOP codon were chosen.

**Table 1:** gRNAs

Target	gRNA name	gRNA sequence (20xN-NGG)
p53 Exon3	p53 gRNA #1 (759)	AGTGAAGCCCTCCGAGTGTC-AGG
p53 Exon3	p53 gRNA #2 (760)	AGGAGCTCCTGACACTCGGA-GGG
p53 Exon3	p53 gRNA #3 (761)	GACACTCGGAGGGCTTCACT-TGG
p63 Exon3	p63 gRNA #1	TCCACAAAGTTCAACTCGAT-GGG
p63 Exon4	p63 gRNA #2	CCGTCACGCTATTCTGTGCG-TGG
p63 Exon4	p63 gRNA #3	AGCCCCAGGTTCGTGTACTG-TGG
p73 Exon3	p73 gRNA #1	CCGGGGTAGTCGGTATTGGA-AGG
p73 Exon3	p73 gRNA #2	CGGGGTGTAGGGGCTCGCCG-GGG
Puma Exon1	Puma gRNA #1	ATGGCCCGCGCACGCCAGGA-GGG
Puma Exon1	Puma gRNA #2	AGCTCTCCGGAGCCCGTAGA-GGG
Puma Exon1	Puma gRNA #3	GGAAGGGGCGCGGACTGTCG-CGG
Bim Exon1	Bim gRNA #1	ACTTACATCAGAAGGTTGCT-TGG
Bim Exon1	Bim gRNA #2	TTGCGGTTCTGTCTGTAGGG-AGG
Foxo1 Exon1	Foxo1 gRNA #1	TCGTCGCGCCGCAACGCGTG-GGG
Foxo1 Exon1	Foxo1 gRNA #2	GGAGAGTGAGGACTTCGCGC-GGG
Foxo3a Exon1	Foxo3a gRNA #1	CACGCCGCCACCGATCACCA-TGG
Foxo3a Exon1	Foxo3a gRNA #2	TCTCGATGGCGCGGGTGATC-AGG
Luciferase	Luciferase gRNA	ACCGCTCCGGCGAAGGCGAA-NGG

**Table 2:** Primers

Primer	Sequence (5′-3′)
p53 for	TGTCTGTAAATCCTGCGGGG
p53 rev	GAGGCTAAAAAGGTTCAGGGC
p63 Exon3 for	TAAGACGGTGAGCCACTCCA
p63 Exon3 rev	CCCACTGCAGAAAGCTGAGA
p63 Exon4 for	GATGGGTGGCTTTACTTGGGA
p63 Exon4 rev	ACACACCCTGGAACCTGTCT
p73 for	CTACTCACTGTCCAGGTGGC
p73 rev	ACAAGTAGTGGCCTGTTGGG
Puma for	TTCCTGGGTGGGAGTGACTT
Puma rev	AGGGACTTCCCACTCGACTT
Bim for	ACGAAATGTAGACGTCCCGC
Bim rev	CCCACAGCCTTGAAACCGAT
Foxo1 for	AACCAGTCCAACTCGACCAC
Foxo1 rev	AAGTTCCCAAACGAGCCCTG
Foxo3a for	GGAGAGAGCAAGAGCCCAAG
Foxo3a rev	GACCCTCCCTTCCCACTTTG
qRT-PCR Puma for	GCCCAGCAGCACTTAGAGTC
qRT-PCR Puma rev	GGTGTCGATGCTGCTCTTCT
qRT-PCR L32 for	TTAAGCGAAACTGGCGGAAAC
qRT-PCR L32 rev	TTGTTGGTCCCATAACCGATG

### Cell culture and treatment

HCT116 *p53*^−/−^, HCT116 *p53*^+/+^ (kindly provided by Bert Vogelstein) and HCT116 (ATCC) were maintained in DMEM containing 10% FCS and Penicillin/Streptomycin (P/S). FL5.12 and BaF3 were maintained in RPMI-1640 with 10% FCS, P/S and 1 µg/l recombinant IL-3. To generate IL-2-dependent lymphoid cells, lymph node cells from three C57/Bl6 mice were isolated and activated by 20 ng/ml PMA and 0.5 µg/ml ionomycin for 48 h in RPMI-1640 with 10% FSC and P/S. The cells were then cultured in the medium containing 100 U/ml recombinant IL-2 for 24 h and then used for experiments. For growth factor withdrawal, cells were washed twice with 50 ml PBS and resuspended in RPMI-1640 with 10% FCS and P/S.

### Flow cytometry

For apoptosis quantification by flow cytometry, BaF3 or FL5.12 were deprived of IL-3 in the presence or absence of CT98014 (0.75 µM) for 18 h or as indicated. Activated lymphocytes were deprived of IL-2 in the presence or absence of CT98014 (0.75 µM) for 22 h. The cells were washed once with Annexin-V Binding Buffer (10 mM HEPES, 150 mM NaCl, 150 µM MgCl, 2.5 mM CaCl_2_) stained for 15 min in the dark with Annexin-V-FITC or Annexin-V-APC in the same buffer. The fraction of Annexin-V-positive cells was measured using a FACS Calibur (BD Bioscience) or FACS LSRII (BD Bioscience).

### Western blotting

Cells were harvested and washed with ice-cold PBS. Cells pellets were lysed for 5 min on ice with lysis buffer (20 mM Tris-HCl pH 7.5, 150 mM NaCl, 1% Triton X-100, 1× protease inhibitor cocktail (Roche), phosphatase inhibitor cocktail 1 (1:50, Sigma-Aldrich), MG132 (20 µM, Alexis Biochemicals)) or by nuclear fractionation as described^6^. Lysates were cleared by centrifugation at 16.100×*g* for 10 min, 4 °C. Protein concentration was determined using Bradford reagent (Bio-Rad). Lämmli buffer was added to 10–100 µg of protein lysate and samples were boiled at 95 °C for 5 min. In some cases, the same amount of lysate was loaded on a second gel to probe for several antibodies. Proteins were separated by SDS-PAGE and transferred to nitrocellulose membranes. The membranes were blocked in 3% dry milk in TBS-Tween20 (0.1%) (TBS-T) and then incubated at 4 °C overnight with the primary antibody diluted in 3% dry milk in TBS-T. The membranes were washed three times with TBS-T, incubated for 1 h with horseradish peroxidase (HRP)-conjugated secondary antibody at room temperature and washed three times with TSB-T. Band visualization was achieved using Super Signal West Pico Chemiluminescent Substrate (Thermo Scientific, Waltham, USA) and the Fusion Solo imaging system (Vilber Lourmat, Eberhardzell, Germany).

### Luciferase reporter assay

293T HEK cells in 24-well plates were transfected with a total amount of 1 µg DNA by Attractene. A renilla luciferase expression plasmid (Promega, Madison, WI, USA) as internal control, a luciferase reporter plasmid driven by a 1.7 kb Puma promotor region (−1200 to +500 from ATG) containing a conserved binding site for FOXO3A (CAAACAAT or mutated to CAGGGAAT)^25^ and FOXO3A-TM (T32A, S253A, S315A) pcDNA3.1 expression plasmid were used in a 1:1:1 ratio. Empty vector was added as needed to keep the total DNA constant. Eight hours after transfection DMSO or CT98014 (0.75 µM) were added. Cells were lysed using 100 µl luciferase-lysis buffer (50 mM Tris-phosphate pH 7.8, 250 mM KCl, 10% Glycerol, 0.1% NP-40), lysates cleared by centrifugation at 16.100×*g* for 5 min, 4 °C. A volume of 10 µl of the lysate was added in two black 96-well plates, renilla buffer (25 mM Tris-phosphate, 100 mM NaCl, 1 mM EDTA, 0.05 mM Coelenterazin) or luciferase buffer (25 mM Tris-phosphate, 10 mM MgSO_4_, 2 mM ATP, 0.05 mM Luciferin) were auto-injected and firefly and renilla luciferase activities were determined by a plate reader.

### Immunoprecipitation

293T HEK cells of a 78 cm² culture plate were transfected by PEI with pECE-FLAG-Foxo3a or pECE-FLAG-FOXO3A-TM (gifts from Michael Greenberg (Addgene plasmid # 8360 and # 8361)^26^) and treated with CT98014 (0.75 µM) 8 h later. The next day, the cells were lysed in 1 ml lysis buffer (20 mM Tris-HCl pH 7.5, 150 mM NaCl, 1% Triton X-100, 1× protease inhibitor cocktail (Roche), phosphatase inhibitor cocktail 1 (1:50, Sigma-Aldrich), MG132 (20 µM, Alexis Biochemicals)). A volume of 20 µl were kept as input control and the remaining lysate was rotated for 2 h with 20 µl FLAG-M2 agarose affinity beads (Sigma Aldrich). Beads were washed three times with lysis buffer for 3 min. Immunoprecipitates were eluted in 50 µl lysis buffer with 3× FLAG® peptide (150 ng/µl, Sigma-Aldrich) by rotation for 30 min at 4 °C. Eluates were then subjected to western blotting.

### RT-PCR

Total RNA was extracted using Trizol (Invitrogen, Carlsbad, CA, USA) according to the manufacturer’s protocol. 2 µg RNA were transcribed into cDNA using the SuperScript First Strand Synthesis Kit (Invitrogen). Transcript levels of Puma and L32 were quantified by quantitative RT-PCR on an CFX96TM Real Time System thermocycler (Bio-Rad) with SYBR Green (Eurogentec, Lüttich, Belgium). The following primers were used: mPuma: 5′-GCCCAGCAGCACTTAGAGTC-3′ and 5′-GGTGTCGATGCTGCTCTTCT-3′; mL32: 5′-TTAAGCGAAACTGGCGGAAAC-3′ and 5′-TTGTTGGTCCCATAACCGATG-3′.

### Re-expression of FOXO3A

Production of retroviral particles and infection were performed as described above for lentiviral particles, but using 1.5 µg Hit60, 1.5 µg pVSV-G (Clontech) and either 1.5 µg pLXIN-hFOXO3A, plXIN-hFOXO3A-TM, or pLXIN-emtpy. Only BaF3 expressing lentiCRISPRv2 gRNA #1 targeting mouse *Foxo3a* were used, as human *Foxo3a* is also targeted by mouse *Foxo3a* gRNA #2. Selection was achieved with 50 ng/mL Geneticin™ (ThermoFisher, Waltham, MA, USA). Experiments were performed immediately after successful selection.

### Overexpression of FOXO3A in HCT116 *p53*^−/−^

HCT116 *p53*^−/−^ in six-well plates were transfected by Lipofectamine® 2000 with pECE-FLAG-FOXO3A or empty vector according to the manufacturer’s protocol. Twenty-four hours after transfection, the cells were treated with GDC-0941 (10 µM) for 5 h and then subjected to western blotting.

### Statistical analysis

All data are shown as mean ± S.D. in case of technical replicates and as mean ± 95% confidence interval in case of biological replicates. The GraphPad Prism 5 software (GraphPad Software Inc., La Jolla, CA, USA) was applied for statistical analysis using the one-way ANOVA with post hoc Tukey’s multiple comparison test. *P* ≤ 0.05 was regarded as the threshold value for statistical significance. *P ≤ 0.05; **P ≤ 0.01; ***P ≤ 0.001.

## Electronic supplementary material

Fig.S1

Fig.S2

Fig.S3

Fig.S4

Fig.S5

Supplementary Figure Legends
