# Supplementary material for: Requirement of GSK-3 for PUMA induction upon loss of pro-survival PI3K signaling
Source: Cell Death Dis. 2018 Apr 23;9(5):470. doi: 10.1038/s41419-018-0502-4 (PMC5913275; doi:10.1038/s41419-018-0502-4)
Supplement: Supplementary file 1 — Fig.S1 [file 41419_2018_502_MOESM1_ESM.pptx]

## Slide 1
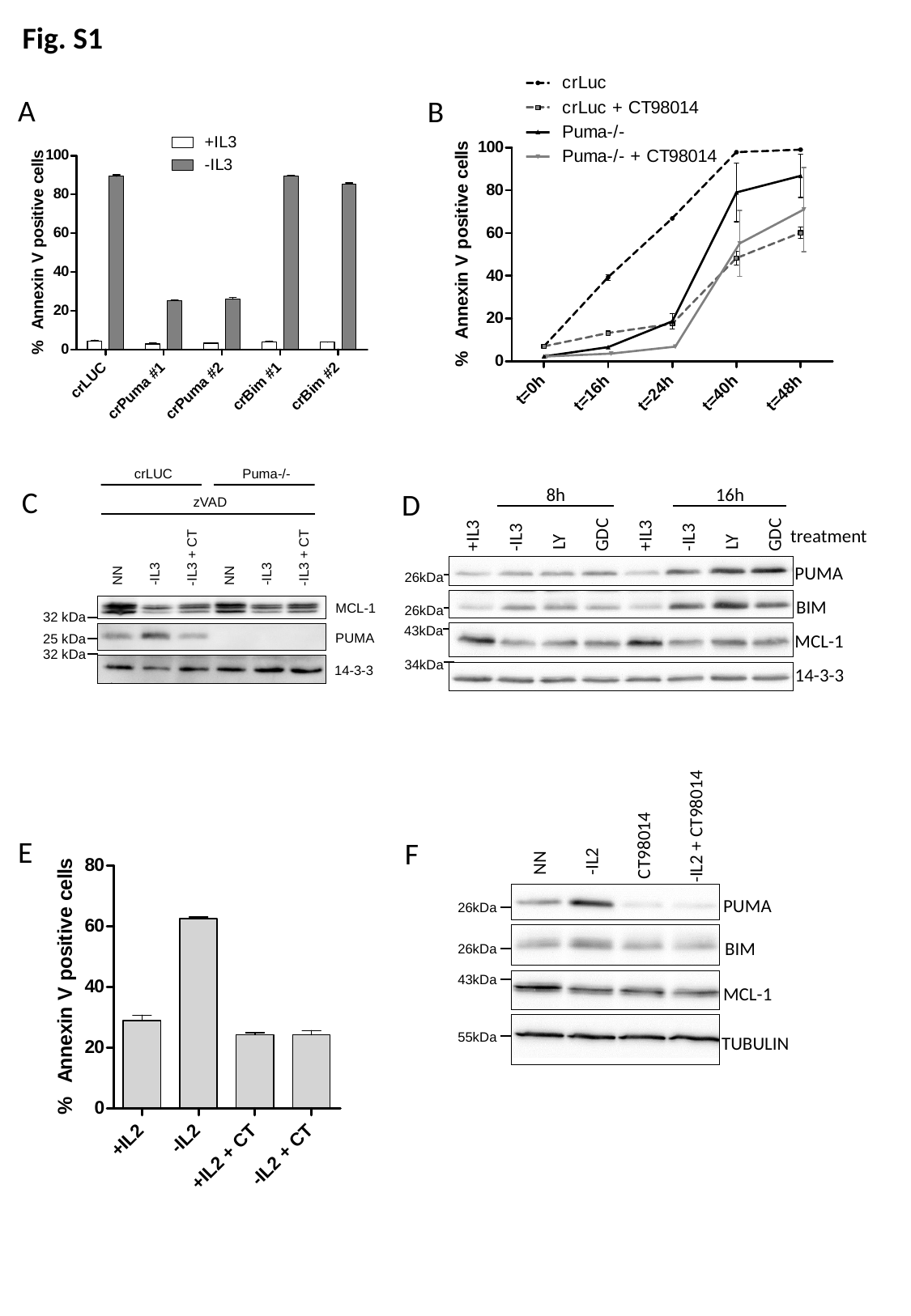

Fig. S1
A
B
crLUC
Puma-/-
8h
16h
C
D
zVAD
treatment
GDC
GDC
+IL3
+IL3
-IL3
-IL3
LY
LY
-IL3 + CT
-IL3 + CT
PUMA
-IL3
-IL3
NN
NN
26kDa
BIM
MCL-1
26kDa
32 kDa
43kDa
MCL-1
PUMA
25 kDa
32 kDa
34kDa
14-3-3
14-3-3
-IL2 + CT98014
E
F
CT98014
-IL2
NN
PUMA
26kDa
BIM
26kDa
43kDa
MCL-1
55kDa
TUBULIN
