# Supplementary figures and images for: Requirement of GSK-3 for PUMA induction upon loss of pro-survival PI3K signaling
Source: Cell Death Dis. 2018 Apr 23;9(5):470. doi: 10.1038/s41419-018-0502-4 (PMC5913275; doi:10.1038/s41419-018-0502-4)

## Slide 1
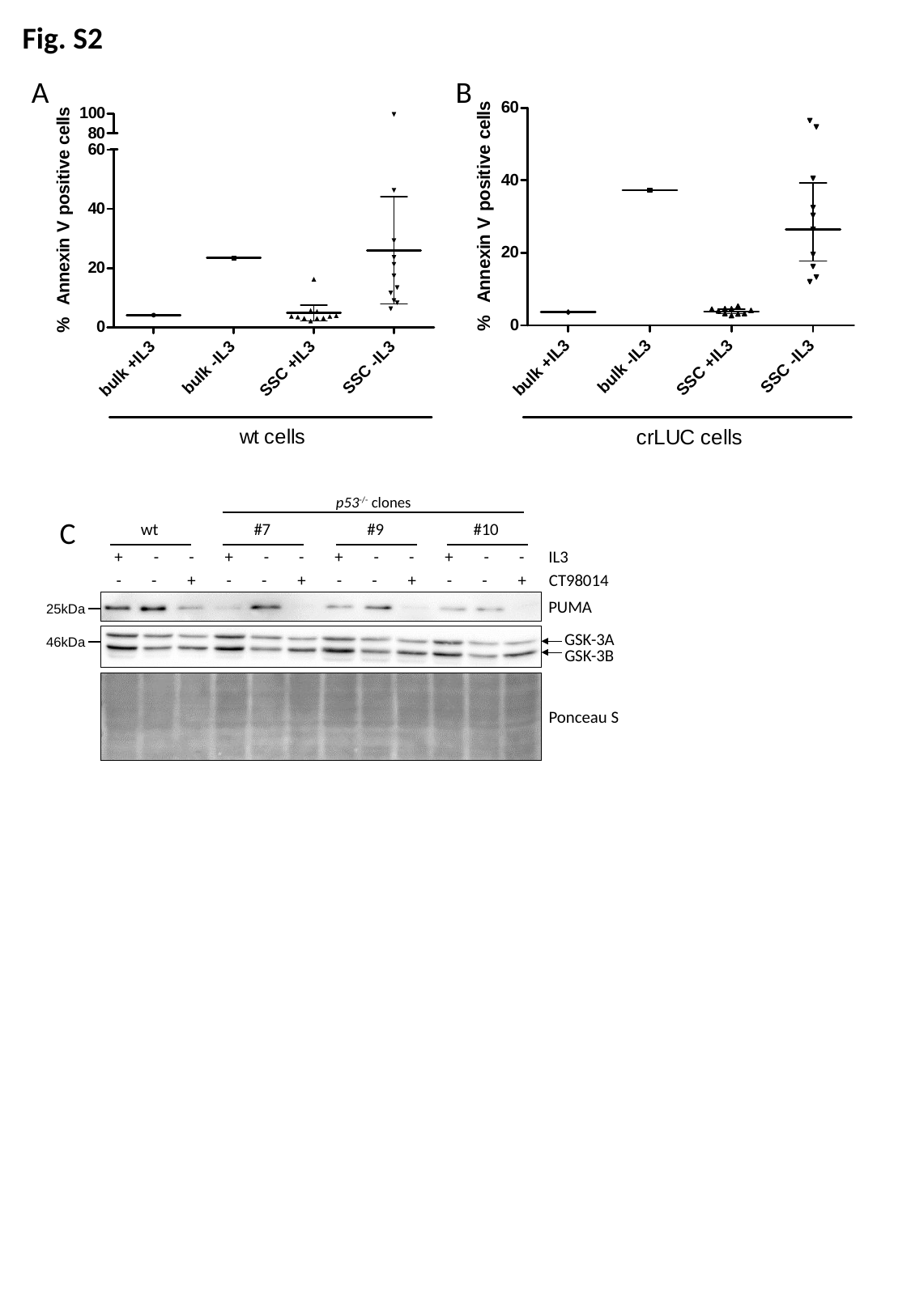

Fig. S2
A
B
p53-/- clones
C
wt
#7
#10
#9
+
-
-
+
-
-
+
-
-
+
-
-
IL3
-
-
+
-
-
+
-
-
+
-
-
+
CT98014
PUMA
25kDa
GSK-3A
46kDa
GSK-3B
Ponceau S

Supplement: Supplementary file 2 — Fig.S2 [file 41419_2018_502_MOESM2_ESM.pptx]

## Slide 1
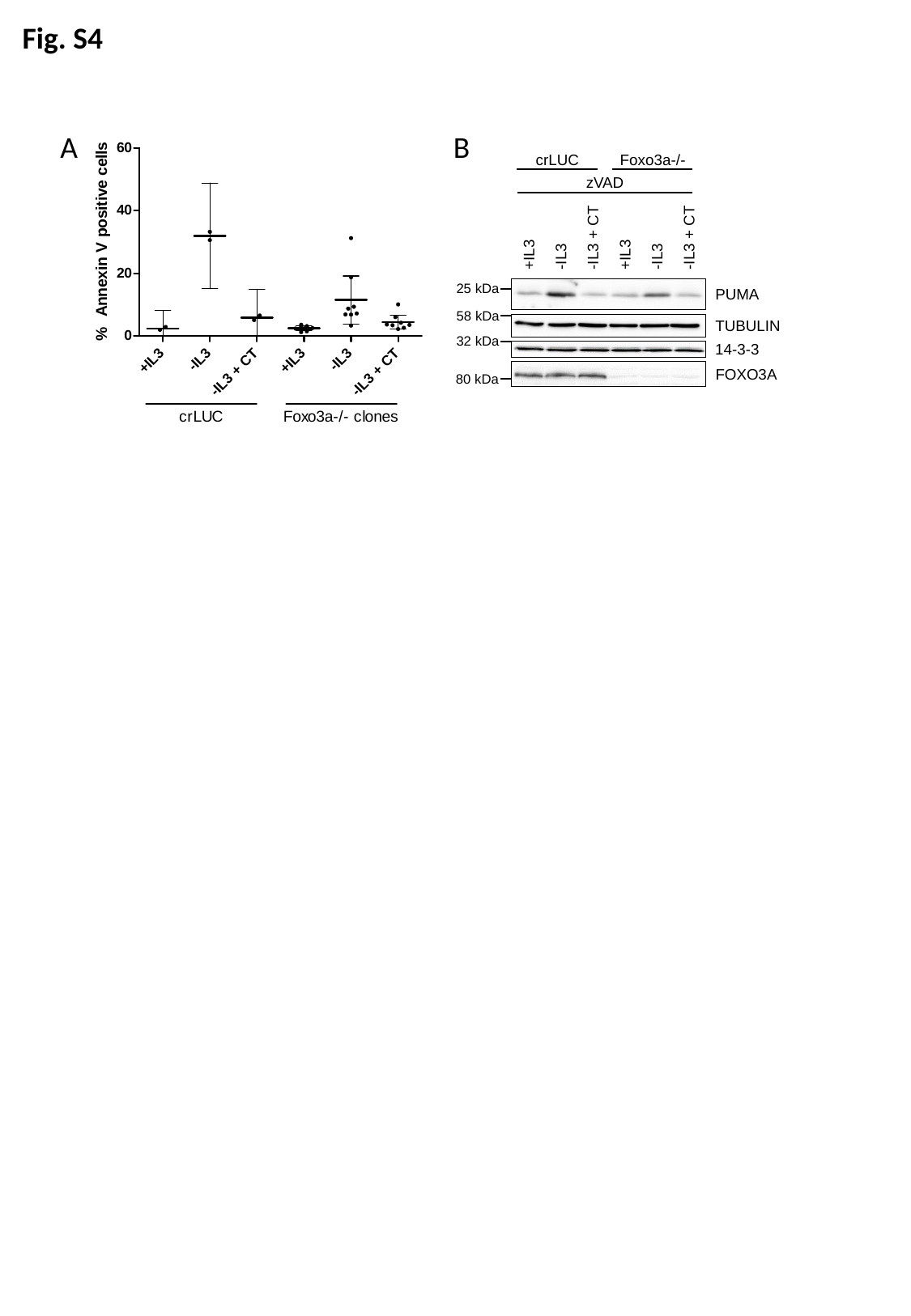

Fig. S4
A
B
crLUC
Foxo3a-/-
zVAD
-IL3 + CT
-IL3 + CT
+IL3
+IL3
-IL3
-IL3
25 kDa
PUMA
58 kDa
TUBULIN
32 kDa
14-3-3
FOXO3A
80 kDa

Supplement: Supplementary file 4 — Fig.S4 [file 41419_2018_502_MOESM4_ESM.pptx]

## Slide 1
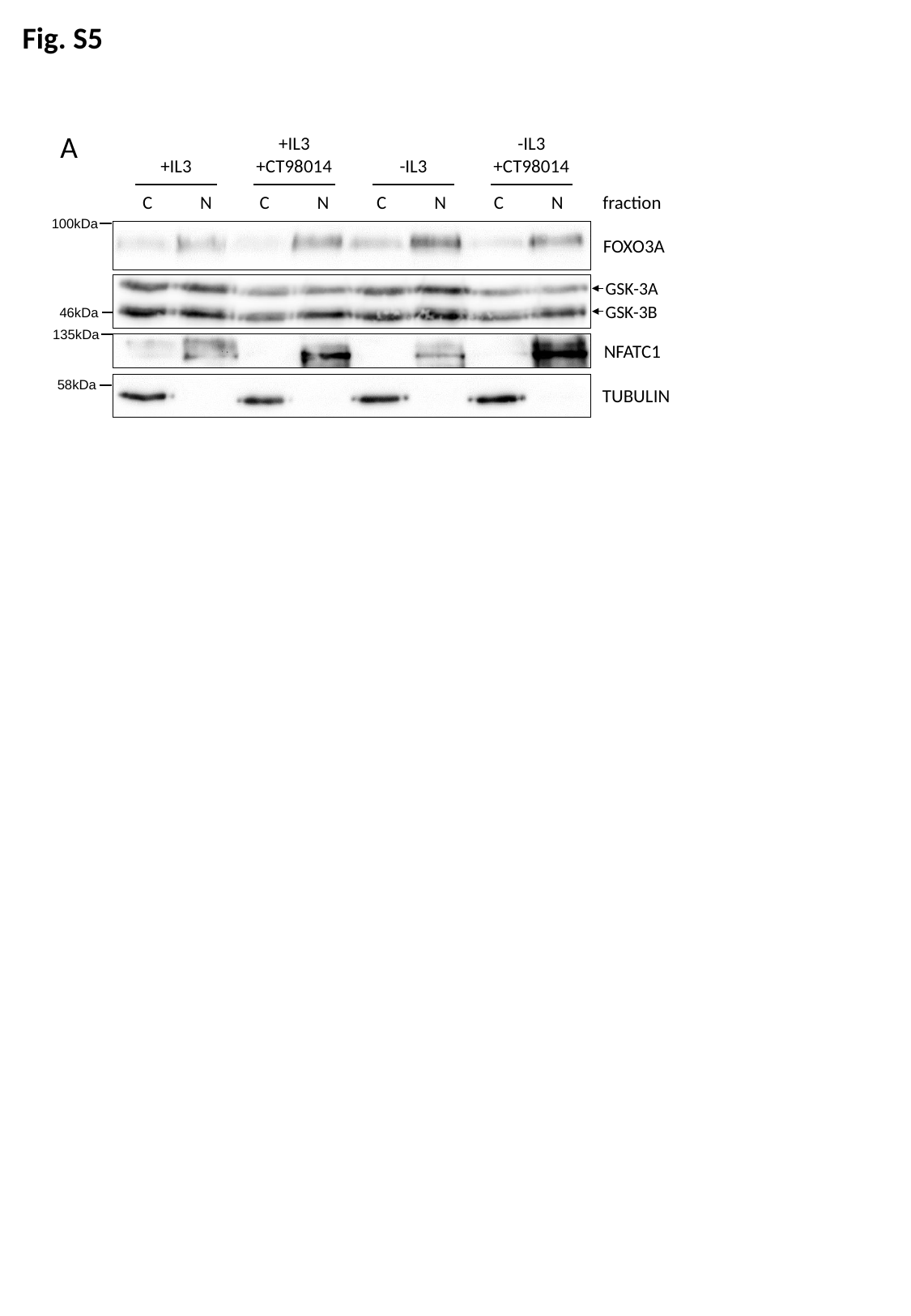

Fig. S5
A
+IL3
+CT98014
-IL3
+CT98014
+IL3
-IL3
C
N
C
N
C
N
C
N
fraction
100kDa
FOXO3A
GSK-3A
GSK-3B
46kDa
135kDa
NFATC1
58kDa
TUBULIN

Supplement: Supplementary file 5 — Fig.S5 [file 41419_2018_502_MOESM5_ESM.pptx]
