# Supplementary material for: Requirement of GSK-3 for PUMA induction upon loss of pro-survival PI3K signaling
Source: Cell Death Dis. 2018 Apr 23;9(5):470. doi: 10.1038/s41419-018-0502-4 (PMC5913275; doi:10.1038/s41419-018-0502-4)
Supplement: Supplementary file 3 — Fig.S3 [file 41419_2018_502_MOESM3_ESM.pptx]

## Slide 1
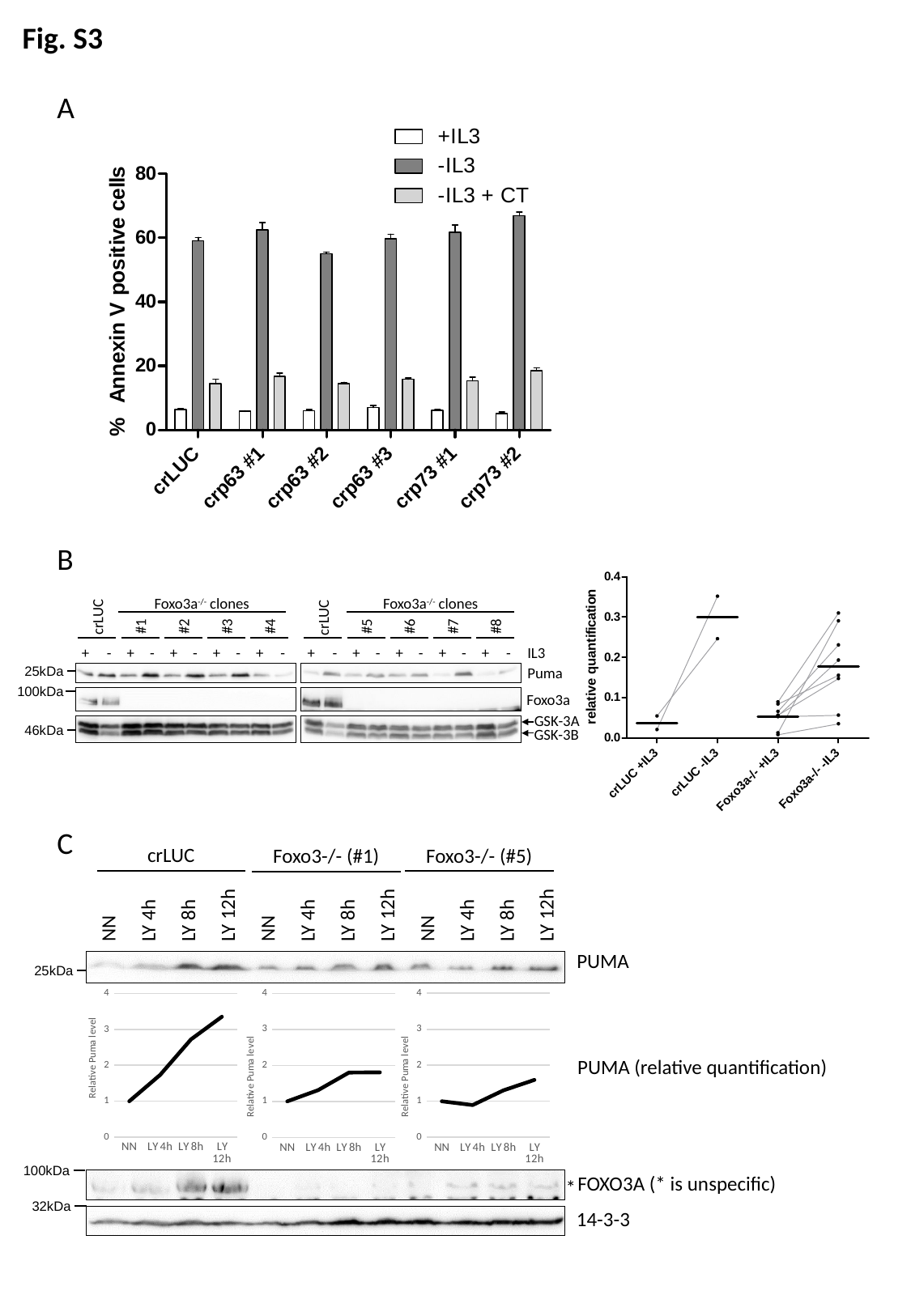

Fig. S3
A
B
Foxo3a-/- clones
Foxo3a-/- clones
crLUC
crLUC
#6
#7
#8
#1
#2
#3
#4
#5
+
-
+
-
+
-
+
-
+
-
+
-
+
-
+
-
+
-
+
-
IL3
25kDa
Puma
100kDa
Foxo3a
GSK-3A
46kDa
GSK-3B
C
crLUC
Foxo3-/- (#5)
Foxo3-/- (#1)
LY 12h
LY 12h
LY 12h
LY 4h
LY 8h
LY 4h
LY 8h
LY 4h
LY 8h
NN
NN
NN
PUMA
25kDa
### Chart
| Category | |
|---|---|
| NN | 1.0 |
| LY 4h | 1.310895958198863 |
| LY 8h | 1.799446749654219 |
| LY 12h | 1.805747656370063 |
### Chart
| Category | |
|---|---|
| NN | 1.0 |
| LY 4h | 0.896231752857305 |
| LY 8h | 1.304288785787032 |
| LY 12h | 1.592735091094263 |
### Chart
| Category | |
|---|---|
| NN | 1.0 |
| LY 4h | 1.732690794649882 |
| LY 8h | 2.719315499606608 |
| LY 12h | 3.349527930763179 |PUMA (relative quantification)
100kDa
FOXO3A (* is unspecific)
*
32kDa
14-3-3
