# Supplementary material for: Requirement of GSK-3 for PUMA induction upon loss of pro-survival PI3K signaling
Source: Cell Death Dis. 2018 Apr 23;9(5):470. doi: 10.1038/s41419-018-0502-4 (PMC5913275; doi:10.1038/s41419-018-0502-4)
Supplement: Supplementary file 6 — Supplementary Figure Legends [file 41419_2018_502_MOESM6_ESM.docx]

**Supplementary Figure Legends**

**Figure S1 - Apoptosis induced by growth factor-withdrawal requires GSK-3 dependent PUMA induction**

1. FL5.12 cells expressing CRISPR/Cas9 constructs, targeting *Luciferase* (crLUC), *Puma* (crPuma) or *Bim* (crBim) were deprived of IL-3 and analyzed for apoptosis by Annexin V staining after 16 h. Error bars represent SD from technical replicates.
2. Ba/F3 cells expressing CRISPR/Cas9 constructs targeting *Luciferase* (crLuc, two independent cell lines) or *Puma* (four independent single cell clones) were deprived from IL-3 in the presence or absence of CT98014 (0,75 µM) and apoptosis was assessed by Annexin-V-FITC staining and flow cytometry after 16, 24, 40 and 48 hours. The 0 h condition represents cells kept in medium with IL-3. Error bars represent SEM from biological replicates.
3. Ba/F3 cells expressing CRISPR/Cas9 constructs targeting *Luciferase* or a pool of Puma^-/-^ single cell clones (Clones #1-8) were deprived of IL-3 in the presence or absence of CT98014 (0.75 µM, CT) and treated with zVAD (20 µM) for 16 h. Cell lysates were analyzed by Western blot using antibodies as indicated.
4. Ba/F3 wild type cells were deprived of IL-3 or treated with the PI3K inhibitors LY294002 (10 µM) or GDC-0981 (10 µM) for 8 or 16 h. Cells grown in presence of IL-3 were taken as control. Cell lysates were analyzed by Western blot using antibodies as indicated.
5. Primary, activated IL-2 dependent lymphocytes obtained from C57BL/6 mouse lymph nodes were deprived of IL2 in presence or absence of CT98014 (CT, 0.75 µM) for 22 h. The cells were harvested, stained with Annexin-V-FITC and analyzed by flow cytometry. Error bars represent SD from technical replicates.
6. Cells treated as in (B) were harvested after 13h, subjected to Western blotting and analyzed using antibodies as indicated.

**Figure S2 - A minor role of p53 for GSK-3 dependent PUMA induction**

1. Single cell clones from Ba/F3 wt cells and Ba/F3 wt bulk cultures were deprived of IL-3 for 18 h and subsequently stained with Annexin-V-FITC and analyzed by flow cytometry. Each dot represents the mean of a duplicate measurement from one single cell clone or the bulk culture. Error bars for single cell clones represent 95% confidence interval.
2. Single cell clones from Ba/F3 cells expressing CRISPR/Cas9 constructs targeting *Luciferase* and Ba/F3 bulk cultures expressing CRISPR/Cas9 targeting *Luciferase* were deprived of IL-3 for 18 h and subsequently stained with Annexin-V-FITC and analyzed by flow cytometry. Each dot represents the mean of a duplicate measurement from one single cell clone or the bulk culture. Error bars for single cell clones represent 95% confidence interval.
3. Ba/F3 wt or p53^-/-^ single cell clones (#7, #9, #10) were cultured in the presence or absence of IL-3 or CT98014 (CT, 0.75 µM) for 14 h, subjected to Western blotting and analyzed by Western blotting using antibodies as indicated.

**Figure S3 - FOXO3A is an important PUMA inducer upon growth factor deprivation**

1. Ba/F3 cells were infected with lentivirus expressing CRISPR/Cas9 constructs targeting *P63* (gRNA #1: Exon3/isoform 1, gRNA #2 and #3: Exon 4/all isoforms), *P73* (all isoforms) or *Luciferase* and selected by puromycin. After the selection process, the cells were deprived of IL-3 in the presence or absence of CT98014 (CT, 0.75 µM) for 18 h. The cells were harvested, stained with Annexin-V-FITC and analyzed by flow cytometry. Error bars represent SD of technical replicates.
2. Ba/F3 *Foxo3a*^-/-^ single cell clones and two independent cell lines of Ba/F3 expressing CRISPR/Cas9 constructs targeting *Luciferase* were deprived of IL-3 for 16h, harvested and analyzed by Western blotting with the antibodies indicated. Bands were quantified using FusionCapt Advance Solo 4 16.08. PUMA levels were normalized to GSK-3 levels (loading control).
3. Ba/F3 Foxo3a^-/-^ single cell clones and Ba/F3 cells expressing CRISPR/Cas9 constructs targeting *Luciferase* were treated with LY294002 (10 µM) for 4, 8 or 12 h or left untreated. The cells were harvested after the treatment and lysates were subjected to Western blotting. The protein levels were analyzed using antibodies as indicated. Puma levels were quantified using FusionCapt Advance Solo 4 16.08 and normalized to the untreated control, separately for each cell line.

**Figure S4 – Apoptosis and PUMA induction in the absence of FOXO3A is dependent on GSK-3**

1. Ba/F3 *Foxo3a*^-/-^ single cell clones and two independent cell lines of Ba/F3 expressing CRISPR/Cas9 constructs targeting *Luciferase* were deprived of IL-3 for 16h in the presence or absence of CT98104 (0.75 µM, CT) and analyzed for apoptosis by Annexin-V staining.
2. Pooled Ba/F3 *Foxo3a*^-/-^ single cell clones and Ba/F3 expressing CRISPR/Cas9 constructs targeting *Luciferase* were deprived of IL-3 for 16h in the presence of zVAD (20 µM) and in the presence or absence of CT98014 (0.75 µM, CT).

**Figure S5 - FOXO3A subcellular localization is not influenced by inhibition of GSK-3**

1. Ba/F3 cells were deprived of IL-3 in the presence of absence of CT98014 (0.75 µM) for 5 h. The cells were then subjected to subcellular fractionation into nuclear (N) and cytosolic (C) fractions. Analyzed was performed by Western blotting using antibodies as indicated.
